# Neuroblastoma cells undergo transcriptomic alterations upon dissemination into the bone marrow and subsequent tumor progression

**DOI:** 10.1002/ijc.31053

**Published:** 2017-10-04

**Authors:** Fikret Rifatbegovic, Christian Frech, M. Reza Abbasi, Sabine Taschner‐Mandl, Tamara Weiss, Wolfgang M. Schmidt, Iris Schmidt, Ruth Ladenstein, Inge M. Ambros, Peter F. Ambros

**Affiliations:** ^1^ Department of Tumor Biology Children's Cancer Research Institute (CCRI) Vienna Austria; ^2^ Neuromuscular Research Department Medical University of Vienna Vienna Austria; ^3^ Department of Pediatrics Medical University of Vienna Vienna Austria

**Keywords:** neuroblastoma, disseminated tumor cells, RNA‐Seq, bone marrow

## Abstract

Neuroblastoma is the most common extracranial solid tumor in childhood. The vast majority of metastatic (M) stage patients present with disseminated tumor cells (DTCs) in the bone marrow (BM) at diagnosis and relapse. Although these cells represent a major obstacle in the treatment of neuroblastoma patients, insights into their expression profile remained elusive. The present RNA‐Seq study of stage 4/M primary tumors, enriched BM‐derived diagnostic and relapse DTCs, as well as the corresponding BM‐derived mononuclear cells (MNCs) from 53 patients revealed 322 differentially expressed genes in DTCs as compared to the tumors (*q* < 0.001, |log_2_FC|>2). Particularly, the levels of transcripts encoded by mitochondrial DNA were elevated in DTCs, whereas, for example, genes involved in angiogenesis were downregulated. Furthermore, 224 genes were highly expressed in DTCs and only slightly, if at all, in MNCs (*q* < 8 × 10^−75^ log_2_FC > 6). Interestingly, we found the transcriptome of relapse DTCs largely resembling those of diagnostic DTCs with only 113 differentially expressed genes under relaxed cut‐offs (*q* < 0.01, |log_2_FC|>0.5). Notably, relapse DTCs showed a positional enrichment of 31 downregulated genes on chromosome 19, including five tumor suppressor genes: *SIRT6*, *BBC3*/*PUMA*, *STK11*, *CADM4* and *GLTSCR2*. This first RNA‐Seq analysis of neuroblastoma DTCs revealed their unique expression profile in comparison to the tumors and MNCs, and less pronounced differences between diagnostic and relapse DTCs. The latter preferentially affected downregulation of genes encoded by chromosome 19. As these alterations might be associated with treatment failure and disease relapse, further functional studies on DTCs should be considered.

## Abstract 2

**What's new?**

More than 90% of patients diagnosed with stage 4 metastatic (4/M) neuroblastoma present with disseminated tumor cells (DTCs) in the bone marrow (BM). Despite treatment, a substantial fraction of these patients experience disease relapse. Here, sequencing analysis of tumor tissue, BM‐derived mononuclear cells (MNCs), and DTCs from stage 4/M neuroblastoma patients indicates that numerous genes are differentially expressed in DTCs but are not or are only slightly altered in tumors and MNCs. Moreover, DTCs exhibited significant downregulation of tumor suppressor genes specifically on chromosome 19. Further studies are needed to determine whether DTC transcriptomic alterations are associated with neuroblastoma relapse.

**Abbreviations**
BM: bone marrow
cnLOH: copy‐neutral loss of heterozygosity
DTC: disseminated tumor cell
IF: immunofluorescence
MNA: *MYCN* amplification
MNC: mononuclear cell (in the context of this study: DTC‐depleted MNC fraction of the BM)
MRD: minimal residual disease
OXPHOS: oxidative phosphorylation

## Introduction

Neuroblastoma is the most common solid tumor diagnosed in the first year of life. It is characterized by a remarkably diverse clinical behavior ranging from spontaneous regression or maturation to malignant disease. This diversity is mainly due to the complex biology and genetics of the tumor itself.^1^, ^2^ While tumors with a favorable clinical outcome frequently show numerical chromosomal aberrations and only rarely structural ones, neuroblastomas with unfavorable clinical outcome are commonly characterized by segmental chromosomal aberrations and *MYCN* amplifications (MNAs).^1^, ^3^, ^4^ Deep‐sequencing studies have provided additional insights into the genomic landscape of the tumor, revealing that chromothripsis, as well as mutations and deletions of particular genes are associated with high‐risk disease at various frequencies.^5^, ^6^ In addition, efforts have been made to improve our understanding of the transcriptomic landscape of neuroblastomas. These studies mainly analyzed the prognostic value of gene expression‐based classifiers for neuroblastoma, concluding an improved clinical endpoint prediction.^7^, ^8^, ^9^

Although all these genomic and transcriptomic studies have advanced our understanding of the disease, they have mainly focused on primary tumors. However, a common feature in neuroblastoma is the presence of disseminated tumor cells (DTCs) in the bone marrow (BM) of metastatic (M) stage patients. More than 90% of stage M patients present with DTC infiltration in the BM at diagnosis.^10^ Detection of DTCs in the BM of patients who are older than 18 months is of high prognostic importance, as these patients frequently suffer from disease recurrence and poor outcome.^11^ Importantly, BM can be used for the molecular quantification of minimal residual disease (MRD) and outcome prediction.^12^

In our recent studies, we have shown that BM‐derived DTCs are suitable for genomic and transcriptomic analyses.^13^, ^14^ Furthermore, it has been demonstrated that DTCs can be highly informative regarding the identification of the relapse‐seeding clone.^15^ However, little is known about gene expression changes occurring in DTCs upon dissemination and tumor progression. So far, only a single group has performed gene expression profiling of BM‐derived DTCs from 11 neuroblastoma patients by microarray analysis. In this study, Morandi *et al*. mainly focused on the identification of genes differentially expressed between diagnostic DTCs and primary tumors to identify genes which could serve as prognostic markers for high‐risk neuroblastoma patients.^16^

With our study, we aim to shed more light on the transcriptomic landscape of DTCs, which are frequently considered to lead to relapse in various cancers.^17^ To our knowledge, we present the first RNA‐Seq study of disseminated neuroblastoma cells in which their expression profile was characterized by sequencing the mRNA and comparing the expression profiles of primary tumors, BM‐derived DTCs and the corresponding BM‐derived mononuclear cells (MNCs). Our findings may stimulate further functional research projects to decipher the DTCs that frequently cause relapse in neuroblastoma patients.

## Materials and Methods

### Overview of patient samples

**Figure 1:**
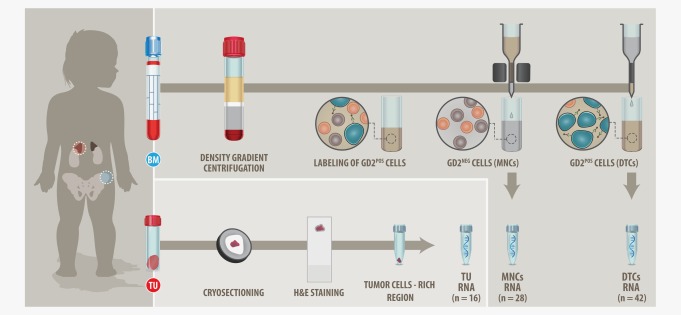
Study design. mRNA of BM cells (DTCs and MNCs) and primary tumors of stage M neuroblastoma patients was sequenced. The MNC fraction of BM aspirates was isolated by density gradient separation, DMSO frozen and stored in liquid nitrogen. Upon thawing, dead cells were removed by an additional density gradient separation and the MNCs were used for a magnetic bead‐based enrichment of DTCs. mRNA of DTCs (*n* = 42) was isolated and sequenced. mRNA of corresponding tumor cells‐depleted BM samples (MNCs, *n* = 28) was sequenced as well (MNC fractions with total RNA <30 ng or detectable DTCs were excluded from sequencing). Primary tumors were cryosectioned and H&E stained. Regions rich in tumor cells were determined by light microscopy and the corresponding regions were cut out of the frozen tumor pieces and used for RNA isolation and sequencing (*n* = 16). [Color figure can be viewed at wileyonlinelibrary.com]

Primary tumor (*n* = 16), DTC (*n* = 42), and corresponding MNC (*n* = 28) samples of 53 stage M neuroblastoma patients were sequenced and analyzed. For three patients (*n* = 3), matching DTC and tumor samples were available at diagnosis. For five patients (*n* = 5), matching diagnostic and relapse DTCs were available. One DTC sample was sequenced twice, before enrichment (D07r2) and after enrichment (D07r). An overview of patients and samples is shown in Figure 1 and the Supporting Information Table S1. RNA‐Seq data are available on the gene expression omnibus (GEO) repository (accession number GSE94035). This study was approved by the St. Anna Kinderspital Ethics Commission in Vienna, Austria.

### Sample preparation for RNA‐seq

The primary tumor and BM samples were stored in liquid nitrogen. Cryosection slides of tumors were prepared and H&E stainings were performed. Identified tumor cells‐rich regions were cut out from the respective tumor pieces and homogenized in QIAzol (Qiagen) with the gentleMACS Dissociator (Miltenyi). The dimethyl sulfoxide (DMSO) frozen MNC fraction of BM aspirates was thawed and density gradient centrifugation (LymphoprepTM, AXIS‐SHIELD PoC AS) was performed. Following the density gradient centrifugation, DTCs were labeled and enriched at 4°C as described earlier.^13^ In brief, the MNC fraction containing DTCs was collected after density gradient separation and washed with phosphate‐buffered saline (PBS) at 300*g* for 10 min at 4°C. The supernatant was discarded and the cells were resuspended in 2 ml ice‐cold magnetic‐activated cell sorting (MACS) buffer (PBS pH 7.2, 0.5% bovine serum albumin, 2 mM ethylene diamine tetraacetic acid). Thereafter, the cell suspension was incubated at 4°C for 20 min with 2.5 µl of FITC‐labeled anti‐GD2 antibodies (14.18 delta CH2 clone), followed by a 15‐min incubation with 75 µl anti‐FITC magnetic beads (Miltenyi) at 4°C. The MACS sorting was continued at 4°C and the enriched DTC fraction and the corresponding tumor cells‐depleted MNC fraction were separately collected and homogenized in QIAzol.

### RNA extraction

Total RNA was extracted with the miRNeasy Micro Kit (Qiagen) following the manufacturer's protocol. Quantity and integrity of extracted RNA was assessed by the Qubit RNA HS Assay Kit (Life Technologies) and the Experion RNA StdSens Assay Kit (BioRad), respectively.

### Library preparation and RNA‐seq

A 30‐ng of total RNA was used for cDNA synthesis following the NEBNext Ultra RNA Library Prep Kit for Illumina protocol (New England BioLabs) with the Poly(A) mRNA Magnetic Isolation Module (New England BioLabs). After cDNA synthesis, the library was completed in an automated way at the EMBL Genomics Core Facility (Heidelberg, Germany). RNA‐Seq was performed at the Illumina HiSeq 2000 platform. Six samples were pooled per lane and 50 bp‐single‐end reads were generated.

### RNA‐seq gene expression and gene set enrichment analysis

Average raw RNA sequencing yield was 36 million (M) reads per sample (range 12–172 M), of which 16 M reads (44%) mapped uniquely to protein‐coding exons (range 5–74 M) (Supporting Information Fig. S1; Table S2). Reads were mapped with GSNAP v2014‐12–28^18^ (“–maxsearch = 100 –npaths = 1 –max‐mismatches = 1 –novelsplicing = 0”) to human reference GRCh37 and then assigned to Ensembl gene models (build 75) using HTSeq^19^ (“htseq‐count ‐f bam ‐t exon ‐s no”). rRNA genes were removed from the Ensembl gene set before read counting and thus excluded from all subsequent analyses. Known single nucleotide polymorphisms (SNPs) and splice sites for GSNAP were extracted from the database SNP (dbSNP) build 138 and Ensembl GRCh37 build 75, respectively. After read mapping and counting, DESeq2^20^ was used to call differentially expressed genes (nbinomWaldTest, minReplicates = 5, cooksCutoff = 0.7, trim = 0.4). In addition, we used DESeq2 to generate a normalized (function “fpm”, robust = TRUE) and variance‐stabilized (function “vst”) gene expression matrix for import into and further analysis in Qlucore v3.2. All samples used in this study passed internal quality control (QC) checks, including base qualities, mapping rates, duplication rates, and 5′–3′ coverage, which were performed on the basis of FastQC and RSeQC reports.^21^

Gene set enrichment analysis (GSEA) was performed with the xtools.gsea.GseaPreranked module implemented in Broad's javaGSEA stand‐alone desktop application (v2.0.13).^22^, ^23^ Gene sets for GSEA were downloaded from MSigDB 5.0.^22^ Input genes were ranked by DESeq2 *p* values from most to least significant, considering the directionality of change. False discovery rates were empirically assessed by permutation analysis with 1,000 iterations. GSEA command line options included “‐collapse false –mode Max_probe –norm meandiv –scoring_scheme weighted –include_only_symbols true –make_sets true –rnd_seed 149 –gui false –nperm 1000 –set_max 5000 –set_min 5” (all other options default).

The complete RNA‐Seq analysis pipeline (including alignment, QC, differential gene expression analysis, and GSEA) was implemented and run on the workflow management platform Anduril (http://www.anduril.org).^24^

### Positional enrichment of differentially expressed genes

To find chromosomal regions enriched for differentially expressed genes, we used the Perl script “pge.pl” provided by De Preter *et al*.^25^ This method implements a hypergeometric test to test for significant co‐localization of a given query gene set among a given background (or reference) gene set. In Figure 6, the query gene set comprised all genes significantly differentially expressed between diagnosis and relapse DTC samples (*q* ≤ 0.2, |log2FC| ≤ 0.5) and the background gene set contained all genes from Ensembl GCRh37 build 75 except rRNA genes.

### Sample preparation and DNA extraction for qPCR

DNA was extracted from samples of 10 patients (*n* = 10) with the high salt extraction method. For four patients (*n* = 4), the DNA was extracted from the primary tumor, the BM‐derived DTCs and a tumor cell‐free BM or peripheral blood sample. For another four patients (*n* = 4), the DNA was extracted from the primary tumor and the BM‐derived DTCs. For the remaining two patients (*n* = 2), the DNA was extracted from DTCs and tumor cells‐free BM.

### Real‐time qPCR

For each quantitative polymerase chain reaction (qPCR) reaction, 6.25 ng DNA was used, and for each sample, three technical replicates were performed. Primers for the nuclear *ALB* gene (located on chromosome 4) and for a 344‐bp fragment of the mitochondrial genome (nucleotides m.8151–8494), encompassing the *MT‐TK* gene were used: *ALB* exon 12 (GenBank NM_000477.6) forward: 5′‐AAT GCT GCA CAG AAT CCT TGGT‐3′, reverse: 5′‐TCA TCG ACT TCC AGA GCT GAAA‐3′; MT‐TK forward: 5′‐CGG GGG TAT ACT ACG GTC AA‐3′, reverse: 5′‐TTT TAT GGG CTT TGG TGA GG‐3′). Template DNA was amplified in the presence of each primer (0.5 µM) and reagents of the SensiMix SYBRHi‐ROX Kit (Bioline) in 12.5 µl‐reactions using the Stratagene Mx3005P qPCR system (Agilent Technologies). Carboxy‐X‐rhodamine (ROX) was used as passive reference dye. Cycling conditions: 10 min 95°C, 40 × [15 sec 95°C, 30 sec 60°C, 20 sec 72°C], followed by a dissociation segment for melting curve analysis. SYBR Green fluorescence data were analyzed using the MxPro 4.10 software (Agilent Technologies). The relative mitochondrial DNA (mtDNA) amount for each patient sample was obtained by normalization of the *MT‐TK* Ct values with the Ct values of the *ALB* gene. Ct values were calibrated to the DNA of peripheral blood from a healthy individual.

### Genomic copy‐number analysis

For 34 enriched DTCs, we used one aliquot of the samples for genome analysis that was performed earlier.^13^, ^15^ The data were analyzed with the ChAS software (Affymetrix). SNP array data analyzed for these studies are available on the GEO repository under the accession number GSE84291, and the corresponding GEO identifier can be found in the Supporting Information Table S3.

## Results

### Magnetic bead‐based enrichment led to a three‐fold increase in DTC purity

**Figure 2:**
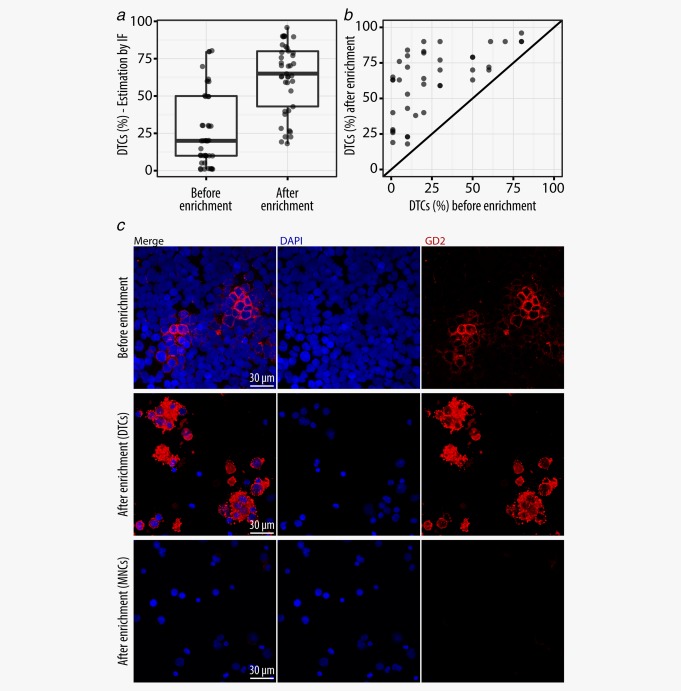
Efficiency of DTC enrichment. (*a*) Tumor cell content of DTC samples before and after enrichment determined by GD2‐IF. (*b*) Enrichment efficiency for each individual DTC sample determined by IF. (*c*) IF staining of BM aspirates before enrichment and corresponding positive (enriched DTCs) and negative (DTC‐depleted MNCs) fractions after enrichment. [Color figure can be viewed at wileyonlinelibrary.com]

DTCs are usually underrepresented in the BM, which necessitates their enrichment before gene expression analysis. The median infiltration of GD2^POS^ tumor cells in the BM aspirates (*n* = 42) was determined by immunofluorescence (IF) microscopy to be 20% before enrichment (range 1%–80%) and 65% (range 19%–96%) after magnetic bead‐based enrichment (Fig. 2 *a*). The DTC content after enrichment was sample‐dependent (Fig. 2 *b*) with 27% of samples containing <50% DTCs, 37% of samples containing 50%–75% DTCs, and 37% of samples with >75% DTCs after enrichment. Overall, enrichment resulted in a threefold increase in tumor cell content within DTC samples. Negative fractions (MNCs) were examined by IF (Fig. 2 *c*) and all samples with detectable GD2^POS^ DTCs were excluded from further analysis.

### Gene expression signature dominated by cell type and *MYCN* expression

**Figure 3:**
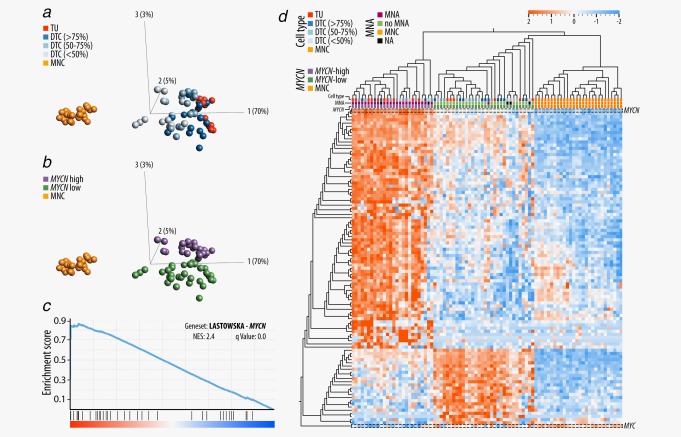
Cell type and *MYCN* driven expression signatures of tumor, DTC and MNC samples. (*a*) Principle component analysis of TU, DTC and MNC samples with cell type specific annotations. DTC samples were annotated in three different groups, according to the tumor cell content determined by IF. (*b*) Principle component analysis of TU, DTC and MNC samples with a *MYCN* expression level specific annotation. (*c*) A representative gene set (LASTOWSKA_COAMPLIFIED_WITH_MYCN) that was enriched in the comparison of DTC *MYCN*‐high and DTC *MYCN*‐low samples. (*d*) Unsupervised clustering of TU, DTC and MNC samples based on the expression of 101 genes: the top 100 differentially transcribed genes between DTC *MYCN*‐high and DTC *MYCN*‐low samples (sorted by *q* value, |log_2_FC|>1) and in addition *MYC*. *MYCN* is highlighted in the heat map as the most significantly upregulated gene in the DTC *MYCN*‐high samples (*q* = 3.7 × 10^−51^, log_2_FC = 4.9). *MYC* is highlighted, but is not among the top 100 differentially expressed genes (*q* = 0.047, Log_2_FC = −1). Abbreviation: NES: normalized enrichment score. [Color figure can be viewed at wileyonlinelibrary.com]

We expected differences in global gene expression patterns to be largely driven by cell type (for convenience we label tumor, DTC and MNC samples as distinct *cell types* in this article). Indeed, principal component analysis of all genes transcribed in tumors and MNCs unambiguously separated samples by cell type along the first principal component (*x*‐axis), with tumor samples (16 diagnostic samples) clustering to the right and MNC samples (*n* = 28: 14 diagnostic samples and 14 relapse samples) to the left (Fig. 3 *a*). In‐between these two clusters and along the same axis, DTCs (*n* = 42: 22 diagnostic and 20 relapse samples) scattered according to their tumor cell content. *MYCN* expression status explained most of the remaining gene expression variability in tumor and DTC samples (second principal component, *y*‐axis) (Fig. 3 *b*), revealing a strong effect of *MYCN* upregulation on the global gene expression of tumors (*n* = 6 with *MYCN*‐low and *n* = 10 with *MYCN*‐high) and DTCs (*n* = 26 with *MYCN*‐low and *n* = 16 with *MYCN*‐high). GSEA for DTC *MYCN*‐high and *MYCN*‐low samples revealed several previously described *MYCN*‐associated gene sets (Supporting Information Table S4), including a gene set containing genes coregulated with *MYCN* upregulation in primary neuroblastomas^26^ (Fig. 3 *c*). Furthermore, unsupervised clustering with the top‐100 differentially expressed genes between *MYCN*‐high and *MYCN*‐low DTC samples (Supporting Information Table S5) separated tumors and DTCs by *MYCN* expression rather than cell type or tumor cell content (Fig. 3 *d*). Seventy six genes were upregulated in DTC *MYCN*‐high samples, with *MYCN* itself being the most significantly upregulated gene (*q* = 3.7 × 10^−51^, log_2_FC = 4.9). Except for one case (D04d, sample with 82% tumor cells), *MYCN* expression levels were in line with the MNA status as determined by fluorescence in situ hybridization (FISH) and/or SNP array (Supplementary Information Table S1, with additional information for D04d). D04d and the matching tumor sample contained approximately 25–30 copies of *MYCN* as determined by SNP array (Supporting Information Fig. S2). Unfortunately, no expression information on the corresponding tumor was available due to degraded RNA in this sample. The remaining 24 genes were downregulated in the DTC *MYCN*‐high samples. Although not among the top‐100 regulated genes, *MYC* was downregulated in DTC *MYCN*‐high samples (*q* = 0.006, log_2_FC = 1).

### Candidate markers for MRD diagnosis in stage M neuroblastoma patients

**Figure 4:**
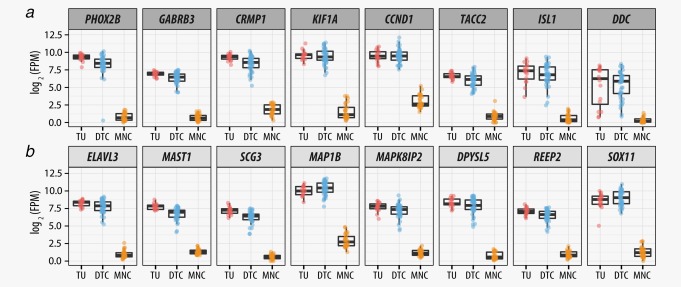
Differentially expressed genes in tumor, DTC and MNC samples. (*a*) Expression of MRD marker in the BM of neuroblastoma patients that are known from the literature.^27^ (*b*) The top eight upregulated genes in DTCs as compared to the corresponding MNCs. Abbreviation: FPM: fragments per million. [Color figure can be viewed at wileyonlinelibrary.com]

The identification of robust MRD markers is a crucial step in the exact diagnosis of tumor cells in the BM. By comparing the gene expression of DTCs (*n* = 42) and MNCs (*n* = 28), we found that eight markers for MRD diagnosis in neuroblastoma proposed by Cheung *et al*.^27^ were upregulated in tumor and DTC samples, and only marginally or not at all transcribed in the corresponding MNC samples (Fig. 4 *a*). Among these eight genes, *PHOX2B* was the most significantly upregulated gene in our data set (*q* = 3 × 10^−234^, log_2_FC = 8.7). Noteworthy, in one DTC sample (D27d), *PHOX2B* was not transcribed. *GABRB3*, *CRMP1*, *KIF1A*, *CCND1*, *TACC2*, *ISL1* and *DDC* were all significantly upregulated in DTCs as compared to MNCs (*q* < 8.5 × 10^−74^, log_2_FC > 6). However, it is notable that *CCND1* was also transcribed by non‐tumor cells of the BM. Furthermore, we found a higher variation in the *DDC* expression among tumor and DTC samples as compared to the other genes. This variance was associated with *MYCN* expression as *DDC* was downregulated in DTC *MYCN*‐high (*n* = 16) samples as compared to *MYCN*‐low samples (*n* = 26) (*q* = 0.03, log_2_FC = 1). In addition to the eight published genes, we identified 224 genes that were significantly upregulated in DTCs as compared to corresponding MNCs (*q* < 8 × 10^−75^ log_2_FC > 6) (Supporting Information Table S6). The eight most significantly upregulated genes (*q* < 1 × 10^−232^) were *ELAVL3*, *MAST1*, *SCG3*, *MAP1B*, *MAPK8IP2*, *DPYSL5*, *REEP2* and *SOX11* (Fig. 4 *b*). The differential expression between DTCs and MNCs of the top seven identified genes was more significant (*q* < 1.3 × 10^−243^) than the differential expression of *PHOX2B (q* = 3 × 10^−234^).

### DTCs overexpress genes encoded by mitochondrial DNA

**Figure 5:**
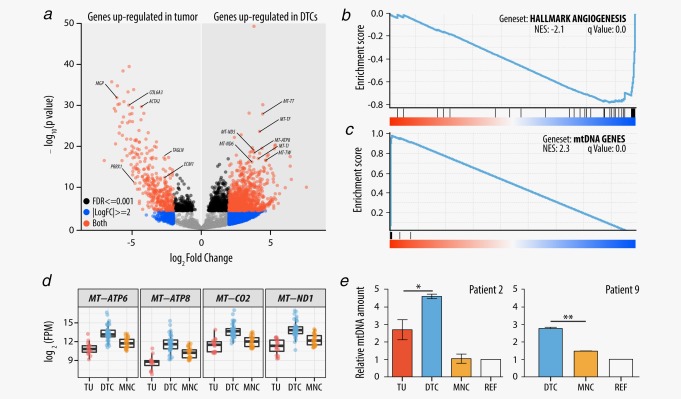
Differentially expressed genes between tumor and DTC samples. (*a*) Volcano plot of log_2_FC *versus p* values comparing gene expression of tumor and DTC samples. *p* values were capped at 1 × 10^−200^. Selected upregulated genes in the tumor are known drivers of metastasis initiation and or angiogenesis. A selection of genes that are encoded by the mtDNA and overexpressed in DTCs is labeled. (*b*) The HALLMARK_ANGIOGENESIS gene set which was enriched in the gene set enrichment analysis for tumor and DTC samples. (*c*) Gene set enrichment analysis for tumor and DTC samples using the complete set of genes encoded by the mitochondrial genome. (*d*) Expression levels of representative genes encoded by the mtDNA in tumor, DTC and MNC samples. (*e*) Relative amount of mtDNA in two representative patients. The mitochondrial DNA amount was determined by qPCR for each patient and with three technical replicates for each sample. Ct values were normalized with Ct values of the *ALB* gene (chromosome 4). All values were calibrated with the mitochondrial DNA quantity of a peripheral blood sample of a healthy individual. REF refers to BM‐derived MNCs of the particular patient at a time point when the BM was tumor cell‐free. Abbreviations: FPM: fragments per million; NES: normalized enrichment score. [Color figure can be viewed at wileyonlinelibrary.com]

The tumor microenvironment is of critical importance for dissemination, arrest and progression of DTCs, and therefore, we were keen to characterize the DTCs which were infiltrating the BM. We compared the expression signature of tumors (total number = 16: 10 with *MYCN*‐high and 6 with *MYCN*‐low) to DTCs (total number = 42: 16 with *MYCN*‐high and 26 with *MYCN*‐low) in a non‐paired manner and found that the expression levels of neuroblastoma hallmark genes *MYCN* and *PHOX2B* were retained in DTCs. However, we identified 322 genes with an altered gene expression (*q* < 0.001, |log_2_FC|>2). We confirmed our observations by comparing the tumor samples with a subset of highly enriched DTC samples (tumor cells > 90%, *n* = 3, Supporting Information Table S7). Among the top differentially expressed genes between tumor and DTC samples (Fig. 5 *a*), we found genes involved in metastasis initiation and angiogenesis, for example, *MGP*, *BGN*, *POSTN* and *ANGPT2*, to be upregulated in tumors. The differentially expressed genes were enriched in several gene sets (Supporting Information Table S8) including the Hallmark Angiogenesis gene set (Fig. 5 *b*) and other gene sets containing genes involved in metastasis initiation. When comparing the gene expression signature of DTC and tumor samples under consideration of the *MYCN* expression level (Supporting Information Table S9), the expression profile largely resembled the above mentioned gene sets.

Interestingly, transcript levels of genes encoded by the mtDNA were found to be significantly higher in DTCs as compared to the analyzed tumors and MNCs (Figs. 5 *a*, 5 *c* and 5 *d*). In a cohort of eight patients, we performed qPCR experiments to quantify the mtDNA levels. In six of these eight patients, the quantity of mtDNA was significantly higher in the DTC samples as compared to the matching tumors (Fig. 5 *e*; Supporting Information Table S10). However, genes encoded by the nuclear DNA (nDNA), which are essential for mitochondrial function such as the oxidative phosphorylation (OXPHOS) and which are part of the same protein complexes as those encoded by the mtDNA, were not upregulated in DTCs (Supporting Information Fig. S3).

### Relapse DTCs downregulate genes on chromosome 19

**Figure 6:**
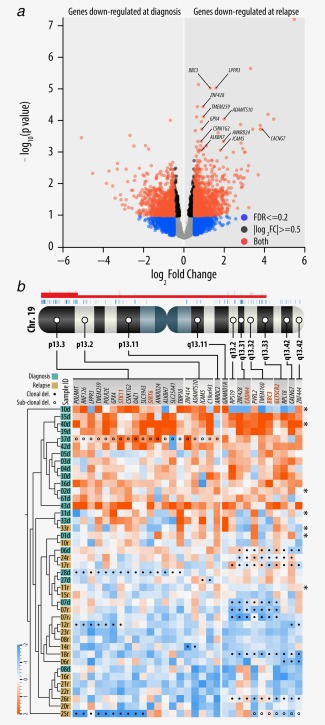
Positional enrichment of genes encoded by chromosome 19. (*a*) Volcano plot of log_2_FC *versus p* values comparing gene expression of diagnostic and relapse DTCs. A selection of genes which are upregulated in relapse DTCs and which are encoded by chromosome 19 is highlighted. (*b*) Ideogram showing the genomic location of 31 differentially expressed genes encoded by chromosome 19. Significantly enriched chromosomal regions (*p* < 0.01) are shown as red segments above the chromosome. Clonal deletions of particular genes are highlighted with solid circles in the heat map. Sub‐clonal deletions are labeled with non‐solid circles. Names of tumor suppressor genes are highlighted. Samples with no available SNP array data are highlighted with (*). [Color figure can be viewed at wileyonlinelibrary.com]

By comparing the gene expression signature of diagnostic (total number = 22: 10 with *MYCN*‐high and 12 with *MYCN*‐low) and relapse DTCs (total number = 20: 6 with *MYCN*‐high and 14 with *MYCN*‐low) in a non‐paired manner, we found the gene expression to be fairly stable over time, with only 113 differentially expressed genes under relaxed cut‐offs (*q* < 0.01, |log_2_FC| > 0.5; Fig. 6 *a*; Supporting Information Table S11). Interestingly, among the downregulated genes in relapse DTCs, we found a strong positional enrichment on chromosome 19 (31 of 113 genes; positional enrichment *q* value = 9 × 10^−38^; Fig. 6 *b*). Notably, we identified five tumor suppressors among the 31 downregulated genes; *STK11*, *SIRT6*, *CADM4*, *BBC3* (also known as *PUMA*) and *GLTSCR2*. Genomic analysis of 34 DTC samples revealed clonal deletions affecting a portion of the 31 downregulated genes encoded by chromosome 19. Although these deletions occurred at higher frequency in relapse patients (9 of 17 samples) as compared to the diagnostic samples (4 of 17 samples), the positional distribution of downregulated and deleted genes did not coincide. While chromosomal aberrations in relapse samples were mainly found on 19q (8 of 20 samples), most of the downregulated genes were encoded by 19p (Fig. 6 *b*; Supporting Information Table S3) which rarely contained deletions (3 of 20 samples). The three tumor suppressor genes encoded by 19q (*CADM4*, BBC3/*PUMA* and *GLTSCR2)* were more frequently deleted in relapse DTCs (29%) as compared to the diagnostic DTCs (12%), whereas the two tumor suppressor genes encoded by 19p were deleted at lower frequencies. By correlating the gene expression of the identified tumor suppressor genes with the overall survival and event free survival (EFS) in publically available datasets^5^, ^28^, ^29^, ^30^ (R2: Genomics Analysis and Visualization Platform http://r2.amc.nl), we found low expression levels of *CADM4* and *BBC3/PUMA* to correlate with worse EFS (Supporting Information Fig. S4; Table S12).

## Discussion

BM is a frequent homing organ for DTCs of various cancer types. It can serve as a metastatic niche for DTCs, which finally can cause disease recurrence after treatment.^31^ Thus, the presence of DTCs is considered as an important prognostic marker for poor outcome in various cancer types, such as breast cancer,^32^ Ewing sarcoma,^33^ and neuroblastoma—with the exception of stage MS tumors which frequently show a minor BM involvement despite mostly favorable disease outcome.^3^, ^34^ Therefore, the characterization of DTCs may help to improve our knowledge about treatment failure and disease relapse.

We searched for expression changes occurring during the course of disease by analyzing the gene expression profiles of diagnostic and relapse DTCs. Unexpectedly, the transcriptional landscape of diagnostic DTCs resembled that of relapse DTCs to a large extent, although these patients had experienced multimodal treatment. This observation may be explained by the protective BM niche, just as it has been shown that the BM microenvironment promotes their survival, dormancy, growth and drug resistance.^35^ Remarkably, we found that the genes which were differentially expressed between diagnostic and relapse DTCs show a positional enrichment on chromosome 19, with five tumor suppressor genes being downregulated in relapse DTCs. Although chromosome 19 deletions are only rarely observed in neuroblastoma,^36^ several studies reported deletions or copy neutral loss of heterozygosity (cnLOH) in primary tumors at low frequency.^37^, ^38^ Interestingly, our recent study revealed a higher frequency of partial chromosome 19 deletions in DTCs of relapse patients as compared to diagnostic DTCs.^15^ Although most reported chromosome 19 deletions in our and other studies affected the *q*‐arm, we found most of the differentially expressed genes to be encoded by the p‐arm. While the downregulation of genes located on the *q*‐arm can be explained, at least partially, by deletions of the particular genes, the rare gene deletions in the p‐arm suggest other, trans‐regulatory mechanisms of gene expression.^1^ Thus, epigenetic studies focusing on DTCs seem reasonable and could shed more light on the transcriptomic alterations occurring in these cells.

Chromosome 19 encodes several well described tumor suppressor genes whose downregulation is associated with progression in various cancers. We identified five of these tumor suppressor genes to be downregulated in relapse DTCs. *BBC3*/*PUMA* is a critical apoptosis inducer whose expression ablation is oncogenic and can lead to therapeutic resistance.^39^ A recent study demonstrated that *BBC3/PUMA* is the critical determinant of the therapeutic response to p53 activation and subsequent apoptosis induced by Nutlin3a, a cancer therapeutics that is in clinical trial.^40^ A screening study with flubendazole, another compound that exerts anti‐cancer activity, identified neuroblastoma as a potential flubendazole‐sensitive cancer entity. The flubendazole efficacy was increased by nutlin‐3, but the flubendazole‐induced anti‐neuroblastoma effect was significantly impaired upon *BBC3/PUMA* depletion in the analyzed neuroblastoma cell lines.^41^ *SIRT6* is another tumor suppressor that we found to be downregulated in relapse DTCs and whose loss was associated with poor outcome in several cancers.^42^ Analysis of data from the Cancer Genome Atlas database revealed that *SIRT6* was deleted in 20% of analyzed cancers.^43^ The loss and/or downregulation of the remaining three suppressor genes in DTCs of relapse samples, namely *STK11*, *CADM4* and *GLTSCR2*, was also reported to be associated with tumor progression in various cancers. Thus, we assume that the downregulation of the five tumor suppressor genes encoded by chromosome 19 in relapse DTCs represents a possible survival advantage for DTCs.

Furthermore, we found that genes involved in metastasis initiation and angiogenesis were upregulated in the primary tumors in contrast to DTCs and MNCs, which reside in the highly vascularized BM.^44^ Besides the downregulation of these genes, DTCs were particularly characterized by an increased amount of mtDNA and the corresponding transcripts that are encoded by this small genome. Notably, also BM‐derived MNCs had elevated levels of mtDNA transcripts as compared to primary tumors, although significantly less than BM‐derived DTCs. A numerical increase in mitochondria is a characteristic feature of various cancers, and it is well known that mitochondria impart considerable flexibility for cancer cells in harsh environments, such as hypoxic BM.^45^ However, the elevated level of transcripts encoded by mtDNA in DTCs is rather surprising, as these genes code only for a minority of subunits that are necessary for functional mitochondria. The vast majority of subunits, for example, for the OXPHOS complex, is encoded by the nDNA. However, unexpectedly, these genes were not upregulated in DTCs. A possible explanation for this phenomenon is the uptake of functional mitochondria by tumor cells from the BM microenvironment.^46^, ^47^ However, functional studies will be necessary to understand the exact mechanism and causes for elevated mtDNA and increased levels of transcripts encoded by it. Apart from these and other differences, DTCs also retained oncogenic features of the tumor. Particularly, the transcription of *MYCN* was comparable in tumor and DTC samples, as well as the transcription of *MYC*, which was earlier shown to be anti‐correlated with *MYCN* in neuroblastoma.^48^

As the BM is a common homing organ for disseminated neuroblastoma cells, it is widely used for MRD testing during or after cytotoxic treatment.^34^ Besides the standardized immunocytological detection of MRD,^10^ real‐time quantitative polymerase chain reaction approaches are also used for MRD detection.^12^ Our three‐fold enrichment of BM‐derived DTCs and their separation from the corresponding BM‐derived MNCs enabled us to characterize their gene expression profiles and to extend the variety of potential marker for MRD detection in stage M neuroblastoma. Our data indicate that the detection of one or two MRD markers can lead to unreliable results, as the expression of tumor‐specific genes varies between samples and, moreover, a number of genes can also be expressed by non‐tumor cells of the BM. Finally, not all supposed NB‐specific markers are expressed by all DTCs, as we could, for example, show for *PHOX2B*, the widely used MRD marker in neuroblastoma. Thus, it is necessary to consider testing and applying MRD marker panels as suggested by Stutterheim *et al*..^49^ For this purpose, for example, 2 to 3 of the 224 most highly and specifically expressed genes in BM‐derived DTCs might be considered for the design of highly specific and sensitive MRD detection markers. Furthermore, feature selection methods^50^ could be applied to our data to determine a minimal discriminant subset of genes being sufficient to classify presence *versus* absence of DTCs in the BM, serving as the basis to create a diagnostic test with a predefined sensitivity and specificity.

In conclusion, our study demonstrated that disseminated neuroblastoma cells retain some of their tumor's oncogenic features, but in addition undergo transcriptomic changes upon dissemination and cancer progression, particularly, the downregulation of tumor suppressor genes encoded by chromosome 19. These insights can stimulate further functional, and especially epigenetic, studies to decipher those cells that are assumed to be the main cause for treatment failure and disease relapse in high‐risk neuroblastoma patients.

## Supporting information

Supporting Information Figure 1

Supporting Information Figure 2

Supporting Information Figure 3

Supporting Information Figure 4

Supporting Information Table 1

Supporting Information Table 2

Supporting Information Table 3

Supporting Information Table 4

Supporting Information Table 5

Supporting Information Table 6

Supporting Information Table 7

Supporting Information Table 8

Supporting Information Table 9

Supporting Information Table 10

Supporting Information Table 11

Supporting Information Table 12

Supporting Information Legends

Supporting Information Letter
