# Supplementary figures and images for: Neuroblastoma cells undergo transcriptomic alterations upon dissemination into the bone marrow and subsequent tumor progression
Source: Int J Cancer. 2017 Oct 4;142(2):297–307. doi: 10.1002/ijc.31053 (PMC5725737; doi:10.1002/ijc.31053)

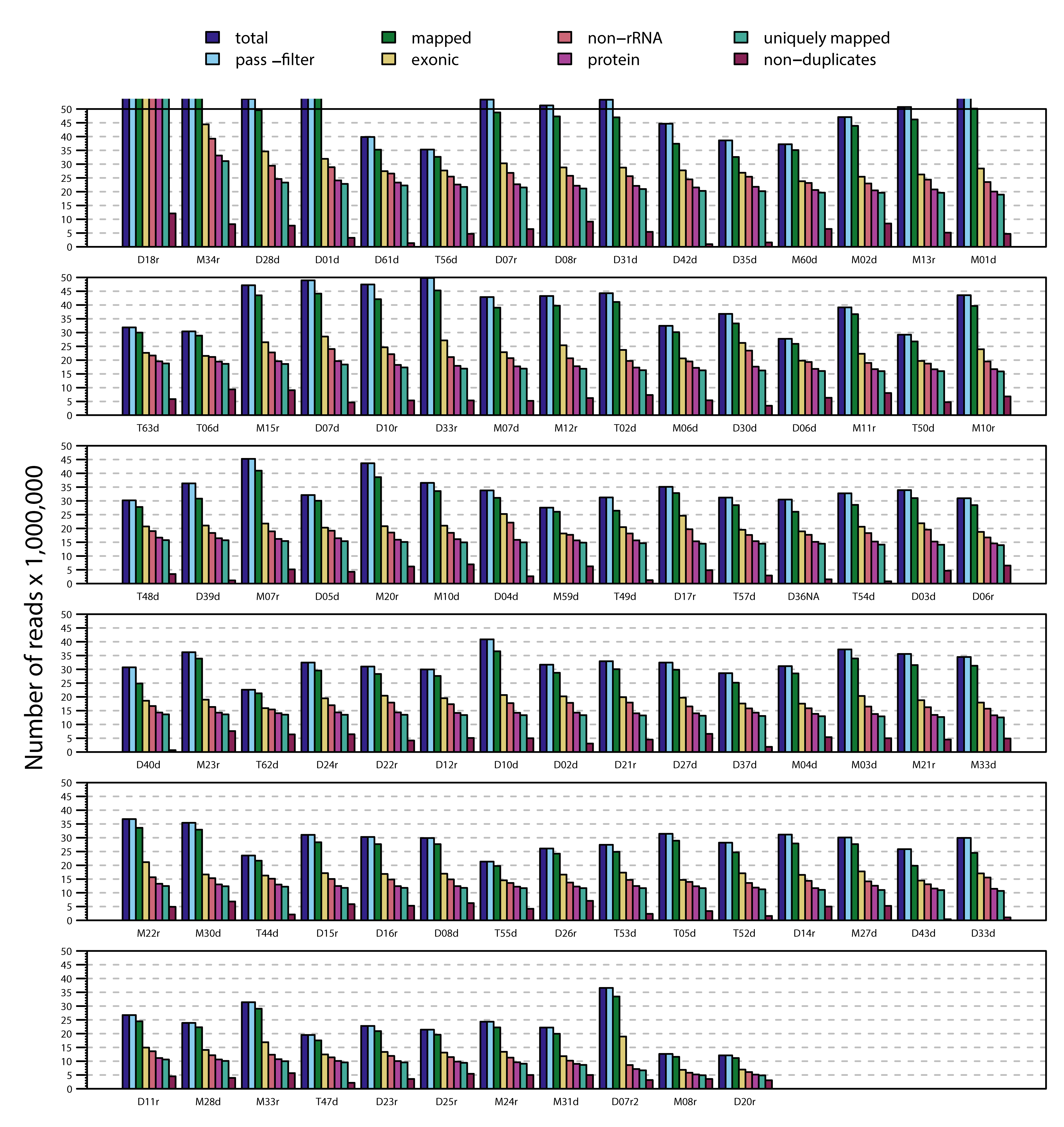

Supplement: Supplementary file 1 — Supporting Information Figure 1 [file IJC-142-297-s001.tif]

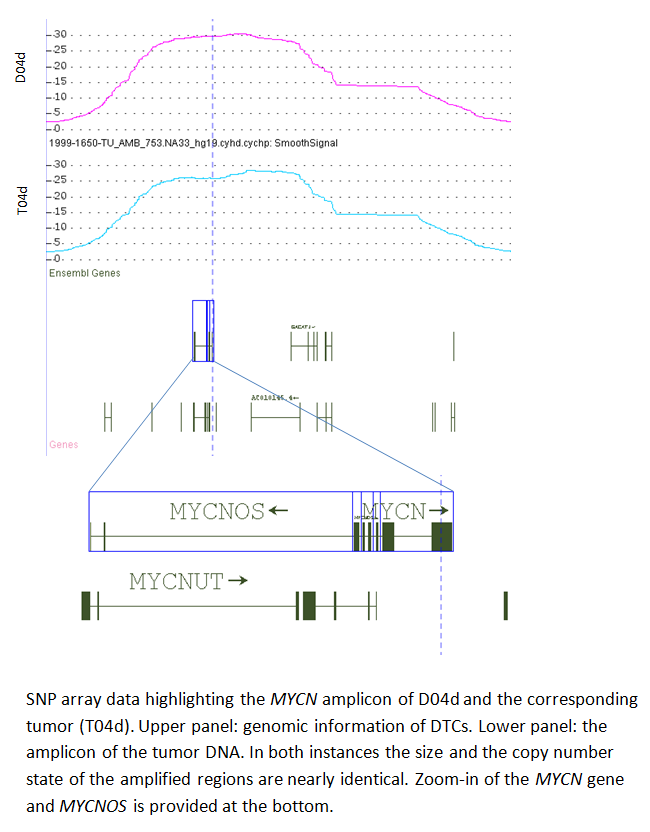

Supplement: Supplementary file 2 — Supporting Information Figure 2 [file IJC-142-297-s002.tif]

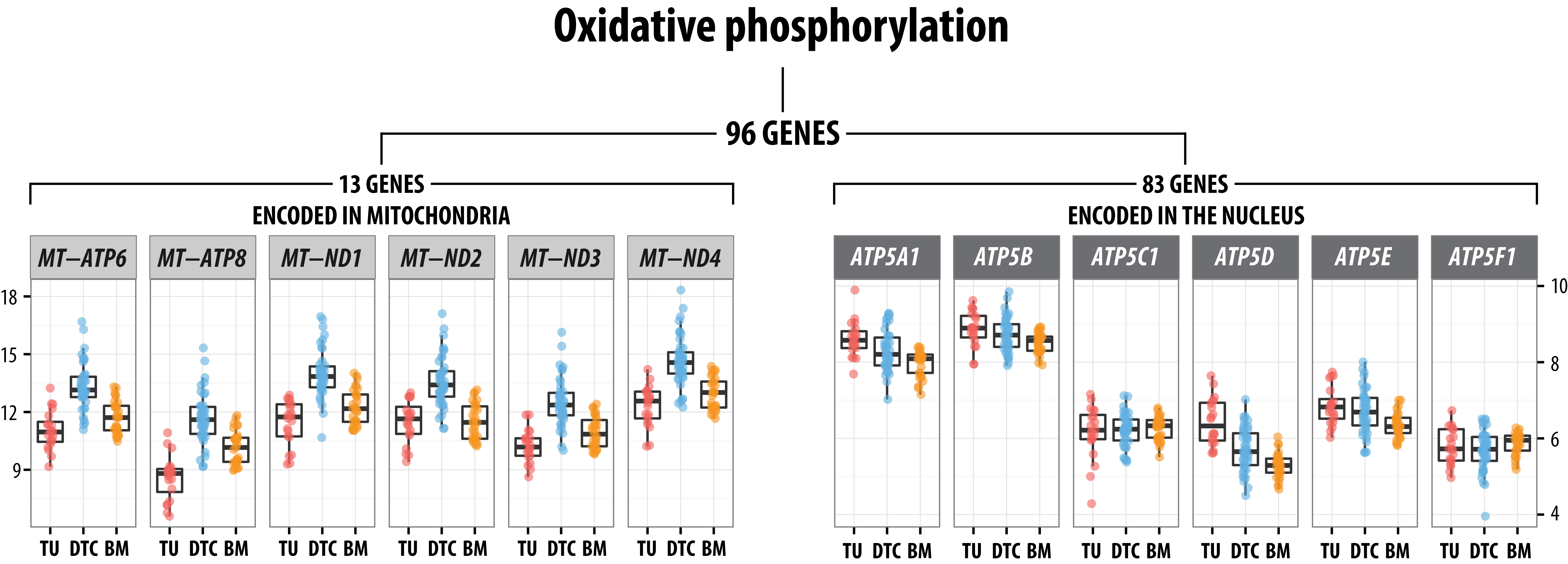

Supplement: Supplementary file 3 — Supporting Information Figure 3 [file IJC-142-297-s003.tif]

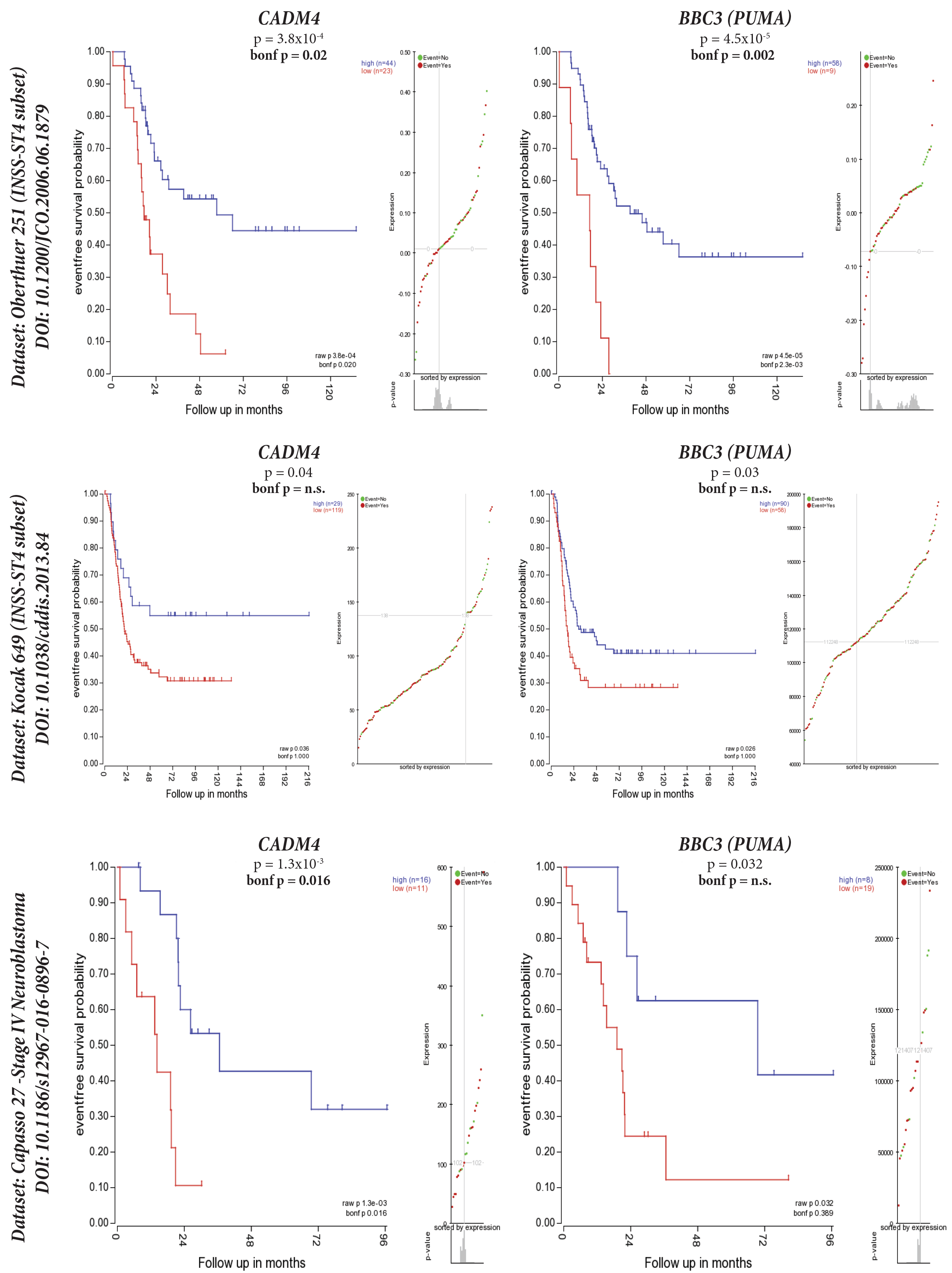

Supplement: Supplementary file 4 — Supporting Information Figure 4 [file IJC-142-297-s004.tif]
