# Supplementary material for: Neuroblastoma cells undergo transcriptomic alterations upon dissemination into the bone marrow and subsequent tumor progression
Source: Int J Cancer. 2017 Oct 4;142(2):297–307. doi: 10.1002/ijc.31053 (PMC5725737; doi:10.1002/ijc.31053)
Supplement: Supplementary file 17 — Supporting Information Legends [file IJC-142-297-s017.docx]

# Supporting Information

## Supporting information figure 1

## File format: Figure (tif)

## Title of data: RNA-Seq reads

Description of data: Overview of RNA-Seq reads among tumor, DTC and MNC samples

## Supporting information figure 2

## File format: Figure (tif)

## Title of data: *MYCN* amplification in sample D04d

Description of data: SNP array data of *MYCN* amplification in sample D04d

## Supporting information figure 3

## File format: Figure (tif)

## Title of data: OXPHOS

Description of data: Differential expression of genes encoded by mtDNA and nDNA and coding for the OXPHOS protein complex

## Supporting information figure 4

## File format: Figure (tif)

## Title of data: EFS

Description of data: Correlation of *CADM4* and *BBC3/PUMA* gene expression with the EFS in three publically available datasets.

## Supporting information table 1

## File format: Excel sheet (xlsx)

## Title of data: Overview of patients and samples

Description of data: Overview of tumor, DTC and MNC samples that have been used for RNA-Seq and data analysis

## Supporting information table 2

## File format: Excel sheet (xlsx)

## Title of data: RNA-Seq quality control

Description of data: Quality control of RNA-Seq data

## Supporting information table 3

## File format: Excel sheet (xlsx)

## Title of data: SNP array data of DTCs

Description of data: Overview of genomic aberrations of chromosome 19 in DTCs and the GEO identifiers for the corresponding SNP array (CEL) files.

## Supporting information table 4

## File format: Excel sheet (xlsx)

## Title of data: GSEA for DTC *MYCN*-high vs *MYCN*-low samples

Description of data: Enriched gene sets for the comparison of DTCs MYCN-high vs DTCs MYCN-low.

## Supporting information table 5

## File format: Excel sheet (xlsx)

## Title of data: Top 100 regulated genes DTCs *MYCN*-high vs *MYCN*-low

Description of data: Top 100 regulated genes comparing DTCs *MYCN*-low vs DTCs *MYCN*-high.

## Supporting information table 6

## File format: Excel sheet (xlsx)

## Title of data: MRD - Differentially expressed genes DTCs vs MNCs

Description of data: Potential marker for MRD diagnosis: significantly differentially expressed genes comparing DTCs vs MNCs

## Supporting information table 7

## File format: Excel sheet (xlsx)

## Title of data: Top 322 differentially expressed genes DTCs vs TU

Description of data: Significantly differentially expressed genes comparing DTCs vs TU

## Supporting information table 8

## File format: Excel sheet (xlsx)

## Title of data: GSEA for DTC vs TU

Description of data: Enriched gene sets for the comparison of DTCs vs tumor

## Supporting information table 9

## File format: Excel sheet (xlsx)

## Title of data: DEG between DTC and tumor samples (MYCN groups)

Description of data: A list of DEG between DTC *MYCN*-high vs TU *MYCN*-high and DTC *MYCN*-low vs TU *MYCN*-low

## Supporting information table 10

## File format: Excel sheet (xlsx)

## Title of data: qPCR with mtDNA

Description of data: Overview of all Ct values for ten (n = 10) patients. The highest fold-change is highlighted in red for each patient.

## Supporting information table 11

## File format: Excel sheet (xlsx)

## Title of data: Top 113 differentially expressed genes DTCdx vs DTCrel

Description of data: Significantly differentially expressed genes in comparison: diagnostic DTCs vs relapse DTCs

## Supporting information table 12

## File format: Excel sheet (xlsx)

## Title of data: OS and EFS - TSG

Description of data: Correlation of the *BBC3/PUMA, CADM4, SIRT6, STK11* and *GLTSCR2* gene expression with the OS and EFS in five publically available datasets.
