# Supplementary material for: Neuroblastoma cells undergo transcriptomic alterations upon dissemination into the bone marrow and subsequent tumor progression
Source: Int J Cancer. 2017 Oct 4;142(2):297–307. doi: 10.1002/ijc.31053 (PMC5725737; doi:10.1002/ijc.31053)
Supplement: Supplementary file 18 — Supporting Information Letter [file IJC-142-297-s018.doc]

Dear Prof. Lichter,

Dear editor and reviewer,

Thank you for sending us the comments on our revised manuscript. With this rebuttal letter we hope to have adequately addressed the three remarks raised by the reviewer.

**Remark 1:**

**On the authors' answer to remark 2: Modification IV and V: What is the result if transcriptomes of DTC and tumors are compared within the MYCN-high and within the MYCN-low subset separately? It would be (more) interesting to see the results for both groups separately.**

Authors: By comparing the gene expression signatures of DTC and TU samples while considering the *MYCN* expression level, we obtained similar results to the original comparison of the complete DTC and tumor sample sets (up-regulation of genes encoded by the mtDNA in DTCs and differential expression of genes involved in angiogenesis and metastasis initiation, e.g. *MGP*, *BGN*, *POSTN* and *ANGPT2* as already been described in the manuscript). Other changes most likely accounted to the *MYCN* expression signature are now included in the supplementary files.

**Modifications performed in the manuscript (concerning remark 1):**

a. **Lines 269-272** of the newly revised manuscript (results paragraph ***DTCs overexpress genes encoded by mitochondrial DNA***) – it was referred to the newly provided supplementary table containing differentially expressed genes for the following two comparisons: i) DTC *MYCN*-high vs TU *MYCN*-high and ii) DTC *MYCN*-low vs TU *MYCN*-low:

Among the top differentially expressed genes between tumor and DTC samples (Fig. 5A), we found genes involved in metastasis initiation and angiogenesis, e.g. *MGP*, *BGN*, *POSTN* and *ANGPT2*, to be up-regulated in tumors. The differentially expressed genes were enriched in several gene sets (Supporting Information Table 8) including the Hallmark Angiogenesis gene set (Fig. 5B) and other gene sets containing genes involved in metastasis initiation. *When comparing the gene expression signature of DTC and tumor samples under consideration of the MYCN expression level (Supporting Information Table 9), the expression profiles largely resembled the above mentioned gene sets.*

b. A new supplementary table (Supporting Information Table 9) is provided which is containing differentially expressed genes (see above) with log2 fold changes, p values and adjusted p values (q values).

**Remark 2:**

**On the authors' answer to remark 3: In the text also add the information on the copy number of MYCN locus in the DTC sample (also as supplemental figure?).**

Authors: The copy number information of *MYCN* in DTCs was provided and the corresponding figure was included as supplementary information.

**Modifications performed in the manuscript (concerning remark 2):**

a. **Lines 227-231** of the newly revised manuscript (results paragraph ***Gene expression signature dominated by cell type and MYCN expression***) – the copy number information was provided for the corresponding tumor and DTC sample.

Except for one case (D04d, sample with 82% tumor cells), *MYCN* expression levels were in line with the *MYCN* amplification (MNA) status as determined by FISH and/or SNP array (Supplementary Information Table 1, with additional information for D04d). *D04d and the matching tumor sample contained approximately 25-30 copies of MYCN as determined by SNP array (Supporting information figure 04).* Unfortunately, no expression information on the corresponding tumor *was* available due to degraded RNA in this sample.

b. The figure which was provided in the previous rebuttal letter and which was suggested by the reviewer to be included as a supplementary figure is now provided in the revised manuscript (Supporting Information Figure 4).

D04d

T04d

SNP array data highlighting the *MYCN* amplicon of D04d and the corresponding tumor (T04d). Upper panel: genomic information of DTCs. Lower panel: the amplicon of the tumor DNA. In both instances the size and the copy number state of the amplified regions are nearly identical. Zoom-in of the *MYCN* gene and *MYCNOS* is provided at the bottom.

**Remark 3:**

**On the authors' answer to remark 6: I think this sentence can be stated more clearly: "... we used DNA to perform qPCR to count for mtDNA amount and found a significant (?: authors should do a statistical test) increased amount of mtDNA in DTC samples of seven patients..."**

Authors: The sentence was stated more clearly. We performed a statistical analysis (Welch Two Sample t-test) for each patient with matching tumor and DTC samples. The p-values are now provided in the supporting information.

**Modifications performed in the manuscript (concerning remark 3):**

a. **Lines 275-279** of the newly revised manuscript (results paragraph ***DTCs overexpress genes encoded by mitochondrial DNA***) – the sentence was clarified:

Interestingly, transcript levels of genes encoded by the mitochondrial DNA (mtDNA) were found to be significantly higher in DTCs as compared to the analyzed tumors and MNCs (Fig. 5A, 5C and 5D). *In a cohort of eight patients we performed qPCR experiments to quantify the mtDNA levels. In six out of these eight patients the quantity of mtDNA was significantly higher in the DTC samples as compared to the matching tumors (Fig. 5E, Supporting Information Table 10).*

b. A statistical test was performed and the resulting p-values were provided in the Supporting Information Table 10.
